# Early antipsychotic treatment in juvenile rats elicits long-term alterations to the adult serotonin receptors

**DOI:** 10.2147/NDT.S158545

**Published:** 2018-06-14

**Authors:** Michael De Santis, Xu-Feng Huang, Chao Deng

**Affiliations:** 1 Antipsychotic Research Laboratory, Illawarra Health and Medical Research Institute, University of Wollongong, Wollongong, NSW, Australia; 2 School of Medicine, University of Wollongong, Wollongong, NSW, Australia

**Keywords:** antipsychotic drug, serotonin, risperidone, olanzapine, aripiprazole, development, juvenile

## Abstract

**Background:**

Antipsychotic drug (APD) prescription/use in children has increased significantly worldwide, despite limited insight into potential long-term effects of treatment on adult brain functioning. While initial long-term studies have uncovered alterations to behaviors following early APD treatment, further investigations into potential changes to receptor density levels of related neurotransmitter (NT) systems are required.

**Methods:**

The current investigation utilized an animal model for early APD treatment with aripiprazole, olanzapine, and risperidone in male and female juvenile rats to investigate potential long-term changes to the adult serotonin (5-HT) NT system. Levels of 5-HT_1A_, 5-HT_2A_, and 5-HT_2C_ receptors were measured in the prefrontal cortex (PFC), caudate putamen (CPu), nucleus accumbens (NAc), and hippocampus via Western Blot and receptor autoradiography.

**Results:**

In the male cohort, long-term changes to 5-HT_2A_ and 5-HT_2C_ receptors were found mostly across hippocampal and cortical brain regions following early aripiprazole and olanzapine treatment, while early risperidone treatment changed 5-HT_1A_ receptor levels in the NAc and PFC. Lesser effects were uncovered in the female cohort with aripiprazole, olanzapine and risperidone to alter 5-HT_1A_ and 5-HT_2A_ receptors in NAc and hippocampal brain regions, respectively.

**Conclusion:**

The results of this study suggest that early treatment of various APDs in juvenile rats may cause gender and brain regional specific changes in 5-HT_2A_ and 5-HT_2C_ receptors in the adult brain.

## Introduction

Antipsychotic drug (APD) prescription and use is rapidly increasing globally, despite a lack of knowledge on the safety and efficacy of APD use on the developing brain.^1^–^10^ Second-generation APDs including aripiprazole, olanzapine, and risperidone are currently commonly being prescribed (mostly off-label) for the treatment of a variety of childhood disorders from mental illnesses, including depression and child-onset schizophrenia,^5^,^11^,^12^ to various behavioral disorders, including autism spectrum disorder.^13^–^15^

While APDs are known to elicit their therapeutic effects predominantly through a strong affinity and subsequent antagonistic mechanism of action on both the dopamine (DA) D_2_ and serotonin (5-HT) 5-HT_1A_ and 5-HT_2A/2C_ receptors, 16–22 both the dopaminergic and serotonergic neurotransmitter (NT) systems have been proven to undergo, and be heavily involved in, numerous critical neurodevelopmental processes during the childhood/adolescent period.^19^,^23^–^29^ Specifically, 5-HT is known to play an early significant and concentration-dependent trophic role in neural development and neurite growth^8^,^26^,^30^–^33^ and then also undergo specific phases of development as a NT system (eg, synaptogenesis and regressive elimination).^8^,^31^

Subsequently, there is the potential that early insult/use of potent APDs at this critical time of neurodevelopment may have the ability to cause long-term alterations to the functionality of the NT systems, including that of 5-HT, in a manner that precedes normal brain functioning.^8^,^27^,^34^,^35^ With alterations to the 5-HT NT system previously linked to changes in both behavioral attributes (including locomotor, anxiety, and depressive-like behaviors) and furthermore negatively correlated to dopamine NT functioning,^36^–^38^ prescription and use of APDs during the critical neurodevelopmental time period have the potential to lead to long-term deficits in brain functioning.^10^,^35^,^39^

Although current clinical investigations have found some benefits to childhood/adolescent APD use in the treatment of the symptomology of various mental illnesses over a short-term time period (1–2 months) and a time period of up to 6 months,^40^–^42^ the potential for the use of potent APDs to cause long-term alterations to adult brain functioning, especially in a clinical setting, is still mostly unknown.^19^,^43^,^44^

Previously completed animal studies investigating the effects of juvenile APD use on the developing brain, including previous studies completed in our laboratory, have found that early treatment of up to 4 weeks can result in various significant long-term changes to behavioral attributes^39^ and immediate alterations to NT pathways including the 5-HT NT system.^45^,^46^ While investigations into the distribution/density of various NT receptor subtypes, including 5-HT_1A_, 5-HT_2A_, and 5-HT_2C_ receptors, have found various immediate alterations following short-term APD treatment,^44^,^45^,^47^ studies investigating the long-term effects of early APD treatment have been found to be limited to the DA NT system.^19^,^35^,^48^

The present study was subsequently conducted in order to investigate the long-term effects of juvenile APD use with aripiprazole, olanzapine, and risperidone on the adult 5-HT NT system in both male and female rats. Specifically, investigations into adult brain levels of 5-HT_1A_, 5-HT_2A_, and 5-HT_2C_ receptors were investigated in cortical, striatal, and hippocampal brain regions via Western Blot and/or receptor autoradiography experiments.

## Materials and methods

### Animals and housing

Timed pregnant Sprague Dawley rats were obtained at gestation day 16 from the Animal Resource Centre (Perth, WA, Australia) and housed in individual cages under environmentally controlled conditions (22°C, light cycle from 7 am to 7 pm and dark cycle from 7 pm to 7 am). Each was allowed ad libitum access to standard laboratory chow diet (3.9 kcal/g: 10% fat, 74% carbohydrate, and 16% protein) and water. The day of birth was considered postnatal day (PD) 0. Pups were sexed on PD14, and then, 96 rats (48 males and 48 females) were weaned on PD20. Rats were housed in individual rat cages with top wire lids, in which they were able to smell and see each other through the lids.

### Drug treatment groups

After weaning and prior to the commencement of drug treatment, all animals were trained for self-administration by feeding them cookie dough (0.3 g) without drugs two times per day for PD18–21. Rats were then randomly assigned to one of the four experimental groups per gender on PD21 (*n*=12/group): 1) aripiprazole (Otsuka, Tokyo, Japan), 2) olanzapine (Eli Lilly, Indianapolis, IN, USA), 3) risperidone (Apotex, Toronto, ON, Canada), and 4) control (vehicle). Drug treatment was carried out in juvenile rats from PD22–50, a time period equivalent to the childhood/adolescent phase in humans.^26^ In order to replicate a clinical setting, a staggered drug treatment pattern was used, where lower APD dosages are slowly increased to a final dosage amount.^49^ Specifically, APD doses were initiated on PD22 at 0.2 mg/kg for aripiprazole, 0.25 mg/kg three times per day for olanzapine, and 0.05 mg/kg three times per day for risperidone and were increased in three steps over the first 7 days of the 4-week treatment period to achieve a final dose on PD28 of 1 mg/kg three times per day for aripiprazole, 1 mg/kg three times per day for olanzapine, and 0.3 mg/kg three times per day for risperidone. Drug treatment was administered orally to each drug treatment group via mixing cookie dough powder (containing cornstarch 37%, sucrose 37%, gelatine 17%, and casein 9%) with a small amount of distilled water until even in consistency. All animals were individually observed for the duration of each treatment to ensure that they completely consumed the cookie dough pellet and thus received a full dosage. Animals in the control group also received an equivalent pellet without the drug. In consideration of a shorter half-life of APDs in rats, and to ensure a consistently high drug concentration in replication of the clinical scenario of oral administration once per day,^50^ APDs were administered three times per day (at 7, 2, and 10 h) with 8±1 hour intervals. The proposed dosages are translated from a clinical setting and within the recommended dosage ranges for the psychiatric treatment of pediatric patients. Dosage calculations are based on the body surface area formula for dosage translation between humans and rats in the US Food and Drug Administration guideline for clinical trials.^42^,^49^,^51^,^52^ The relevant human equivalent dose (HED) is therefore calculated by the following formula: animal dose (mg/kg) × animal Km (6)/human child Km (25) × body weight (Km factor, body weight (kg) divided by body surface area (m^2^), is used to convert the mg/kg dose to a mg/m^2^ dose). Therefore, for an adolescent with an average weight of 40 kg, the utilized dosages for aripiprazole (1 mg/kg in rats) and olanzapine (1 mg/kg in rats) equals to a clinical dosage of 9.6 mg, while risperidone (0.3 mg/kg) equals to a clinical dosage of 2.88 mg, all within a clinically relevant range for adolescent patients. Previous reports have demonstrated that at this dosage amount, aripiprazole drug treatment reaches >90% DA D_2_ receptor occupancy rates in the rat brain,^53^ while olanzapine and risperidone reach 65%–80% DA D_2_ receptor occupancy rates.^54^,^55^ These dosage amounts have also been shown to be physiologically and behaviorally effective in our laboratory, with similar dosages seen to induce weight gain and changes in hypothalamic neuropeptide Y expression in adolescent rats,^56^ while immediate alterations to both DA receptor and 5-HT receptor binding have been reported in juvenile rats.^45^ All experimental procedures were approved by the Animal Ethics Committee, University of Wollongong, Wollongong, NSW, Australia (AE 12/20) and complied with Australian Code of Practice for the Care and Use of Animals for Scientific Purpose (2004).

### Histological procedures

After a maturation period where all animals were monitored regularly and allowed to mature (PD51–105), all rats were sacrificed on PD106 via carbon dioxide asphyxiation. Euthanasia was completed between 9 am and 11.30 am to minimize the potential circadian-induced variation of protein expression. Brain tissue was removed immediately following euthanasia, frozen in liquid nitrogen, and stored at −80°C until analysis. Six brains from each drug treatment group (n=12) were then randomly assigned for Western Blot analyses, and the remaining six brains from each treatment group were then used for receptor autoradiography experiments. Brain regions involved in both serotonergic signaling and the therapeutic actions of APDs, including the prefrontal cortex (PFC), caudate putamen (CPu), nucleus accumbens (NAc), and hippocampus, were dissected in order to detect 5-HT receptor levels.

#### Microdissection (Western Blot analyses)

Tissue from aforementioned brain regions to be used for Western Blot analysis was collected using microdissection puncture techniques, following a standard procedure in our laboratory.^57^–^60^ Briefly, 500 µm thick sections were cut at −14°C using a cryostat (Leica CM1850; Leica Microsystems, Wetzler, Germany) and collected bilaterally using a microdissection puncher on glass slides.

#### Receptor autoradiography

Tissue from brains selected for receptor autoradiography were collected via coronally dissected sections at −18°C into 14 µm using a cryostat (Leica CM1850). Once dissected, sections were thaw-mounted onto poly-l-lysine (Sigma-Aldrich Co., St Louis, MO, USA)-coated glass slides and stored at −20°C. A set of sections from each animal were stained with the 0.5% cresyl violet solution (Nissl staining) and used to confirm the identification of anatomical structures.

### Western Blot analyses

Tissues obtained from individual rats were homogenized in ice-cold homogenizing buffer (9.8 mL of NP-40 cell lysis buffer; Thermo Fisher Scientific, Waltham, MA, USA; 100 µL of β-glycerophosphate; 50 mM; Thermo Fisher Scientific; 33.3 µL of phenylmethane sulfonyl fluoride; 0.3 M; Sigma-Aldrich Co.; and 100 µL of Protease Inhibitor Cocktail; Sigma-Aldrich Co.). All samples were then centrifuged, with the supernatant solution collected and stored at −80°C until required.

DC™ Protein Assays (#500-0114; Bio-Rad Laboratories Inc., Hercules, CA, USA) were completed at *A*_750 nm_ to spectrophotometrically quantify total protein concentrations. A range of sample protein concentrations were pretested in each region (2, 2.5, 4, 5, 6, 7.5, 8, and 10 µg). A total of 10 µg of protein was selected for PFC, CPu, and NAc regions, while 8 µg of protein was selected for the hippocampus as it best fitted the linear range of signal detection for all tested antibodies. Homogenized brain samples containing the aforementioned microgram concentration of protein were then first heated at 95°C in the loading buffer (950 µL of Laemmli buffer; Bio-Rad Laboratories Inc.; and 50 µL of β-mercaptoethanol; Sigma-Aldrich Co.) for 5 minutes to denature the protein, then placed on ice, and centrifuged for 2 minutes at 4°C. The samples were then loaded into 4%–20% Criterion™ TGX™ Precast Gels (Bio-Rad Laboratories Inc.) and underwent electrophoresis in sodium dodecyl sulfate polyacrylamide gel electrophoresis (SDS PAGE) running buffer (100 mL of 10× SDS-PAGE running buffer; Bio-Rad Laboratories Inc.; and 900 mL of distilled water) at 140 V for 70 minutes. Proteins on the gels were then transferred electrophoretically using the Bio-Rad Midi Format 1-D Electrophoresis Systems onto a polyvinylidene difluoride (PVDF) membrane (Bio-Rad Laboratories Inc.) in ice-cold transfer buffer (150 mL of 10× tris/glycine buffer; Bio-Rad Laboratories Inc.; 300 mL of cold methanol, and 1,050 mL of distilled water) at 100 V for 1 hour. In order to detect the proteins of interest, PVDF membranes were incubated in tris-buffered saline–Tween (TBST) (Sigma-Aldrich Co.) solution containing 5% Blotting-Grade Blocker (nonfat dry milk powder) (Bio-Rad Laboratories Inc.) for 1 hour at room temperature (RT). Membranes were then incubated overnight with the primary antibody, including 5-HT_1A_ (1:2,000; #ab85615; Abcam, Cambridge, UK), 5-HT_2A_ (1:1,000; #sc-50397; Santa Cruz Biotechnology Inc., Dallas, TX, USA), and 5-HT_2C_ (1:1,000; #sc-15081; Santa Cruz Biotechnology Inc.), diluted in TBST buffer containing either 1% bovine serum albumin (BSA) (5-HT_1A_) or 1% nonfat dry milk powder (5-HT_2A_ and 5-HT_2C_). Membranes were either washed three times with TBST for 5 minutes (5-HT_1A_ and 5-HT_2A_) and then incubated with horseradish peroxidase (HRP)-conjugated goat antirabbit secondary antibody for 1 hour at RT (5-HT_1A_ – 1:5,000 and 5-HT_2A_ – 1:3,000; EMD Millipore, Billerica, MA, USA) or washed three times for 20 minutes (5-HT_2C_) and then incubated with HRP-conjugated donkey antigoat secondary antibody for 45 minutes at RT (5-HT_2C_ – 1:2,000; Abcam). Secondary antibodies were diluted in TBST buffer containing either 1% BSA (5-HT_1A_) or 1% nonfat dry milk powder (5-HT_2C_ and 5-HT_2A_). Three TBST washes then followed secondary antibody incubation, and proteins of interest were visualized using the Classico Western horseradish peroxidase (HRP) substrates (EMD Millipore) and Amersham Hyperfilm ECL (GE Healthcare Life Sciences, Wauwatosa, WI, USA). Membranes were then re-probed with mouse antiactin polyclonal antibody (1:10,000, #MAB1501; EMD Millipore) and HRP-conjugated rabbit antimouse secondary antibody (1:3,000, #7076; Cell Signaling Technology, Danvers, MA, USA).

Immunoreactive signals were quantified using the GS-800 image densitometry and Quantity One software (Bio-Rad Laboratories Inc.), and the values were corrected based on their corresponding actin levels. For 5-HT_1A_, the band at ~62 kDa was detected and quantified,^61^ while for 5-HT_2A_, the band at ~55 kDa was detected and quantified.^62^,^63^ Furthermore, for 5-HT_2C_, a band at ~55 kDa was detected and quantified.^64^ The β-actin protein was quantified at 46 kDa. Western Blot gels were arranged by gender, in which each gel contained 24 samples (six rats/group × four treatments [ie, three APDs and one vehicle] × one gender). In order to control for variability, all samples were run in duplicate at second gels at the same sample arrangement and the values of each drug treatment group and control corrected based on their corresponding actin levels. Samples from male and female rats were run in different gels. All results were normalized by taking the value of the vehicle group of each gender as 100% to obtain a comparative value.

### Receptor autoradiography and quantification

Experimental procedures for 5-HT_2A_ binding autoradiography were based on those completed and reported previously.^45^,^65^–^67^ 5-HT_1A_ and 5-HT_2C_ binding autoradiography was also completed; however, binding results were too low and thus discounted from further analysis.

#### 5-HT_2A_ receptor binding procedures

Brain sections for 5-HT_2A_ receptor binding were thawed at RT and then preincubated in 170 mM tris buffer (pH 7.4) for 15 minutes. Slides with sections were then incubated for 2 hours with 2 nM [^3^H]Ketanserin (specific activity: 47.3 Ci/mmol; PerkinElmer Inc., Waltham, MA, USA) in 170 mM tris buffer at RT to determine total binding. Nonspecific binding was determined with the addition of 2 µM Spiperone (Sigma-Aldrich Co.) to subsequent sections. Following incubation, sections were washed four times for 2 minutes in ice-cold buffer, dipped in ice-cold distilled water, and then air dried.^66^,^67^

#### Quantification

Following the completion of receptor binding experiments, all slides were exposed to Amersham Hyperfilm ECL for 2–3 months, along with autoradiographic standards ([^3^H] microscales from Amersham), in X-ray film cassettes. Quantitative analysis of binding images was conducted following the relevant exposure time, using the Multi-Analyst image analysis system (Bio-Rad Laboratories Inc.), connected to a GS-800 Imaging Densitometer (Bio-Rad Laboratories Inc.). Optical density measurement was then converted into femtomoles of [^3^H] ligand per milligram of tissue equivalent (TE) by comparing to the standard. Specific binding was calculated by subtracting nonspecific binding from total binding. A set of sections from each animal were stained with the 0.5% cresyl violet solution (Nissl staining), for the purpose of confirmation of anatomical structures. Specific brain regions in this project were identified by reference to the Nissl-stained sections, along with a standard rat brain atlas.^68^

### Statistical analysis

Statistical analysis of collected data was completed with the use of SPSS software (Windows Version 19.0; IBM Corporation, Armonk, NY, USA). Distribution of data was examined through the Kolmogorov–Smirnov test. All normally distributed data were also analyzed by two-way analysis of variance (ANOVA) (gender × treatment). Male and female data sets were then also analyzed separately by one-way ANOVA, followed by post hoc Dunnett’s tests for multiple comparisons between the treatment groups. Data that were not distributed normally were analyzed via the nonparametric Mann–Whitney *U* test. All data were analyzed per investigated brain region. The data were expressed as mean ± standard error of the mean (SEM). Statistical significance was accepted when *P*<0.05.

## Results

### Long-term effects of adolescent APD treatment on 5-HT_1A_ receptor levels

**Figure 1:**
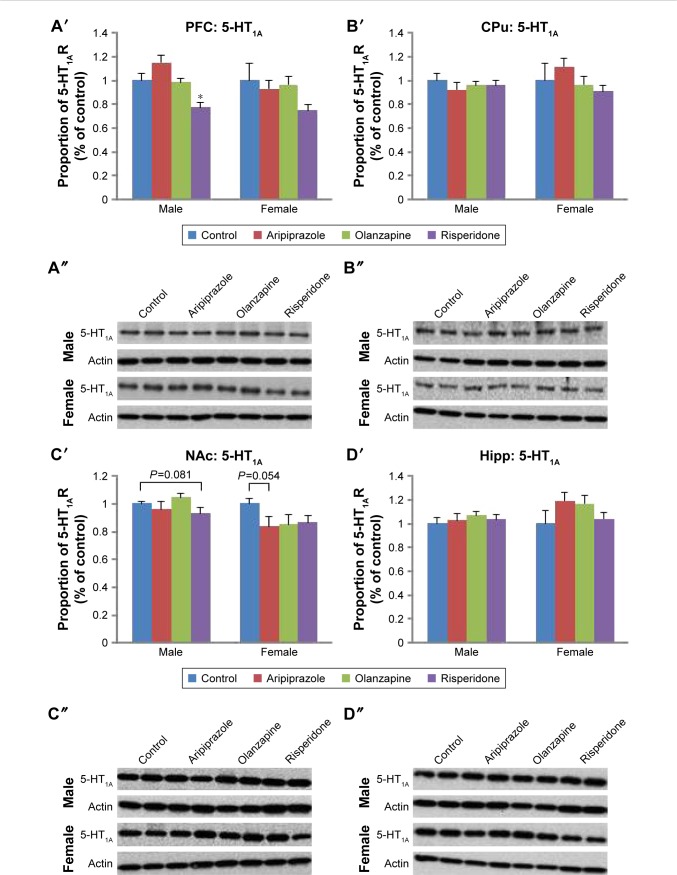
Effects of three APDs on 5-HT_1A_ expression levels in the PFC (**A**′, **A**″), CPu (**B**′, **B**″), NAc (**C**′, **C**″), and Hipp (**D**′, **D**″) of female and male rats. **Notes:** Sprague Dawley rats were treated chronically with aripiprazole (1.0 mg/kg, tid), olanzapine (1.0 mg/kg, tid), risperidone (0.3 mg/kg, tid), or control (vehicle). The number of samples per gender per group is 6. Data were expressed as mean ± SEM. *P<0.05 vs control. The representative bands of Western Blot are shown. **Abbreviations:** APDs, antipsychotic drugs; CPu, caudate putamen; Hipp, hippocampus; 5-HT, serotonin; NAc, nucleus accumbens; PFC, prefrontal cortex; SEM, standard error of the mean; tid, three times daily.

A significant effect of treatment on 5-HT_1A_ receptor protein expression was found in the PFC (*F*_3,45_=4.973, *P*<0.01) and NAc (*F*_3,44_=3.791, *P*<0.02), while a significant effect of gender was also observed in the NAc (*F*_1,44_=13.584, *P*<0.01). Furthermore, a significant interaction between the two factors was found in the CPu (*F*_3,46_=2.860, *P*=0.050) and a trend toward a significant interaction was found in the NAc (*F*_3,44_=2.559, *P*=0.070). Post hoc analysis uncovered that early risperidone treatment decreased 5-HT_1A_ expression in the PFC (−23.8%, *P*<0.02) when compared with the control (Figures 1A′ and A″ and S1). In the male cohort, early APD treatment had a significant effect on the expression of 5-HT_1A_ receptors in the NAc (*F*_3,22_=5.091, *P*<0.01) of adult rats. Further analysis revealed that early risperidone treatment trended to significantly decrease 5-HT_1A_ receptor expression in the NAc (−7.0%, *P*=0.081) (Figures 1 and S1). Analysis of the female cohort found trends toward significant effects of early APD treatment in both CPu (*F*_3,22_=2.853, *P*=0.065) and NAc (*F*_3,20_=2.695, *P*=0.079). Post hoc analysis revealed that early aripiprazole treatment decreased 5-HT_1A_ receptor expression in the NAc (−16.6%, *P*=0.054) when compared with the control group. No significant alterations were uncovered in the CPu (Figures 1B′ and B″ and S1) or hippocampus (Figures 1D′ and D″ and S1) of treated animals in comparison to the control, across either gender.

### Long-term effects of adolescent APD treatment on 5-HT_2A_ receptor levels

**Figure 2:**
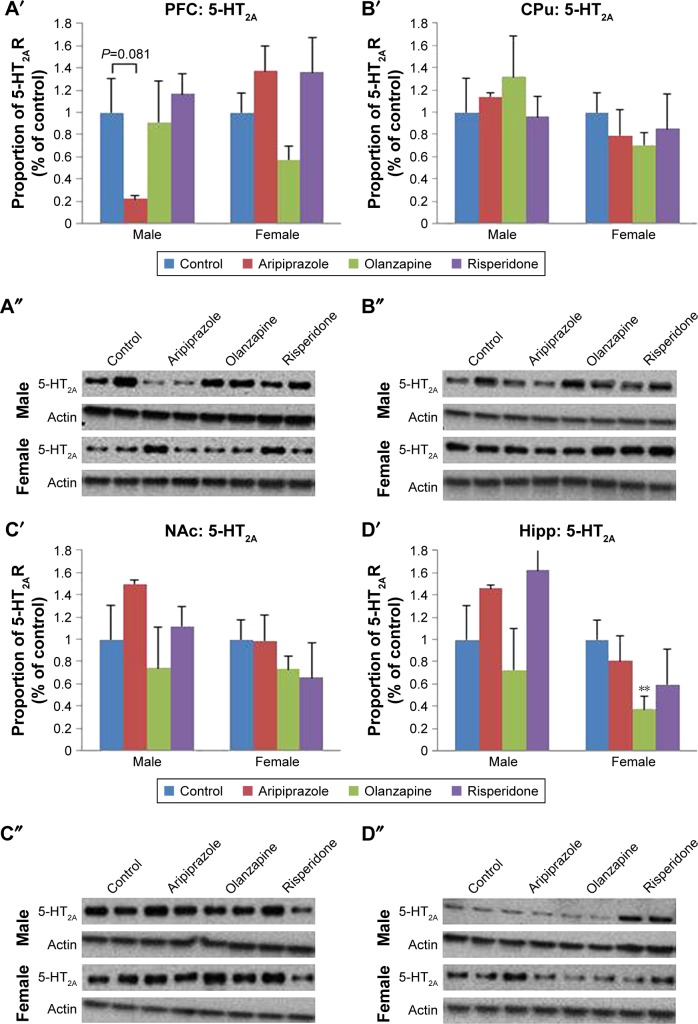
Effects of three APDs on 5-HT_2A_ expression levels in the PFC (**A**′, **A**″), CPu (**B**′, **B**″), NAc (**C**′, **C**″), and Hipp (**D**′, **D**″) of female and male rats. **Notes:** Sprague-Dawley rats were treated chronically with aripiprazole (1.0 mg/kg, tid), olanzapine (1.0 mg/kg, tid), risperidone (0.3 mg/kg, tid), or control (vehicle). The number of samples per gender per group is 6. Data expressed as mean ± SEM. **P<0.01 vs control. The representative bands of Western Blot are shown. **Abbreviations:** APDs, antipsychotic drugs; CPu, caudate putamen; Hipp, hippocampus; NAc, nucleus accumbens; 5-HT, serotonin; PFC, prefrontal cortex; SEM, standard error of the mean; tid, three times daily.

Two-way ANOVA tests (gender × treatment) revealed a significant effect of treatment on 5-HT_2A_ receptor protein expression levels in the hippocampus (*F*_3,45_=4.913, *P*<0.01), while a significant effect of gender was found in the hippocampus (*F*_1,45_=17.745, *P*<0.001) and CPu (*F*_1,46_=4.541, *P*<0.05). Additionally, a significant interaction between the factors was uncovered in the hippocampus (*F*_3,45_=3.340, *P*<0.05) and PFC (*F*_3,46_=3.972, *P*<0.02). Analysis of the male cohort via one-way ANOVA (treatment) uncovered a significant effect of early APD treatment on 5-HT_2A_ receptor expression in the NAc (*F*_3,22_=3.378, *P*<0.05), and hippocampus (*F*_3,22_=4.054, *P*<0.05). Furthermore, a trend to significant effect was also discovered in the PFC (*F*_3,22_=3.035, *P*=0.054). Post hoc analysis discovered that aripiprazole treatment was found to decrease 5-HT_2A_ receptor levels in the PFC (−78.0%, *P*=0.081) upon comparison to the control group. In the female cohort, one-way ANOVA found a significant effect of early APD treatment on 5-HT_2A_ expression in the PFC (*F*_3,22_=3.233, *P*<0.05) (Figures 2A′ and A″ and S2) and hippocampus (*F*_3,21_=4.738, *P*<0.02) (Figures 2D′ and D″ and S2). Further analysis via post hoc tests discovered decreases to 5-HT_2A_ receptor expression in the hippocampus following early olanzapine (−62.4%, *P*<0.01) treatment. No significant changes in the expression of 5-HT_2A_ receptors were uncovered in the CPu (Figures 2B′ and B″ and S2) or NAc (Figures 2C′ and C″ and S2) of APD animals in comparison to the control, across both male and female cohorts.

**Figure 3:**
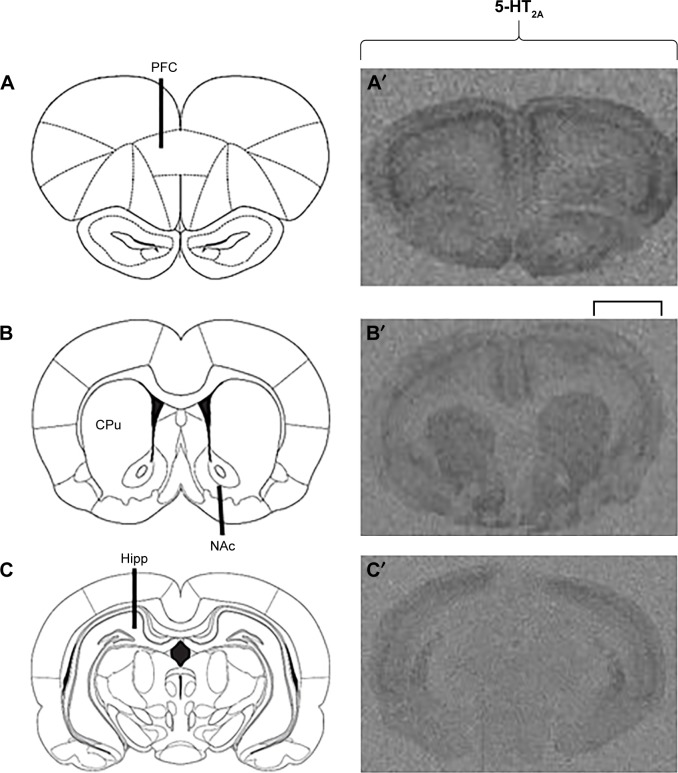
Examples of 5-HT_2A_ receptor binding in the adult rat brain following childhood/adolescent APD treatment. **Notes:** The schematic diagram is adapted from Paxinos G, Watson C. *The Rat Brain in Stereotaxic Coordinates*. 6th edition. London: Elsevier; 2007. © Academic Press 2007.^68^ Showing the level of Bregma for each investigated region (**A**: PFC, 4.68 mm; **B**: CPu and NAc, 1.08 mm; **C**: Hipp, −2.76 mm). (**A**′–**C**′) Examples of autoradiograms to demonstrate [^3^H]Ketanserin binding to 5-HT_2A_ receptors. The scale bar applies to all autoradiograms and is 2.0 mm for A′, 2.8 mm for B′, and 3.2 mm for C′. **Abbreviations:** APD, antipsychotic drug; CPu, caudate putamen; Hipp, hippocampus; 5-HT, serotonin; NAc, nucleus accumbens; PFC, prefrontal cortex.

Examples of [^3^H]Ketanserin binding to 5-HT_2A_ are presented in Figure 3. Detected levels of 5-HT_2A_ in the CPu of females and males, however, were discounted, as expression was too low for accurate quantification. Analysis via two-way ANOVA (gender × treatment) found a significant effect of early APD treatment on the expression of 5-HT_2A_ receptors in the hippocampus (*F*_3,41_=2.106, *P*<0.01), along with a significant interaction between the two factors (*F*_3,41_=1.228, *P*<0.05). A trend to significant effect of treatment was also uncovered in the PFC of rats (*F*_3,43_=4.004, *P*=0.079). Post hoc analysis revealed that early treatment with both aripiprazole (−49.0%, *P*<0.02) and risperidone (−51.1%, *P*<0.01) significantly decreased 5-HT_2A_ expression in the hippocampus in comparison to the control. When subsequently divided by gender, analysis of the male cohort demonstrated a significant effect of early APD treatment on 5-HT_2A_ receptor expression in the PFC (*F*_3,21_=4.010, *P*<0.05) and hippocampus (*F*_3,21_=6.274, *P*<0.01). Further analysis via post hoc revealed that early treatment with aripiprazole decreased 5-HT_2A_ binding in the PFC (−44.3%, *P*=0.064) and hippocampus (−48.8%, *P*<0.05). Similar decreases were also observed following risperidone treatment in the PFC (−60.2%, *P*<0.02) and hippocampus (−69.5%, *P*<0.01) and olanzapine treatment in the hippocampus (−44.7%, *P*=0.063). No significant effects were found in the female cohort between the APD treatment group and control.

### Long-term effects of adolescent APD treatment on 5-HT_2C_ receptor levels

**Figure 4:**
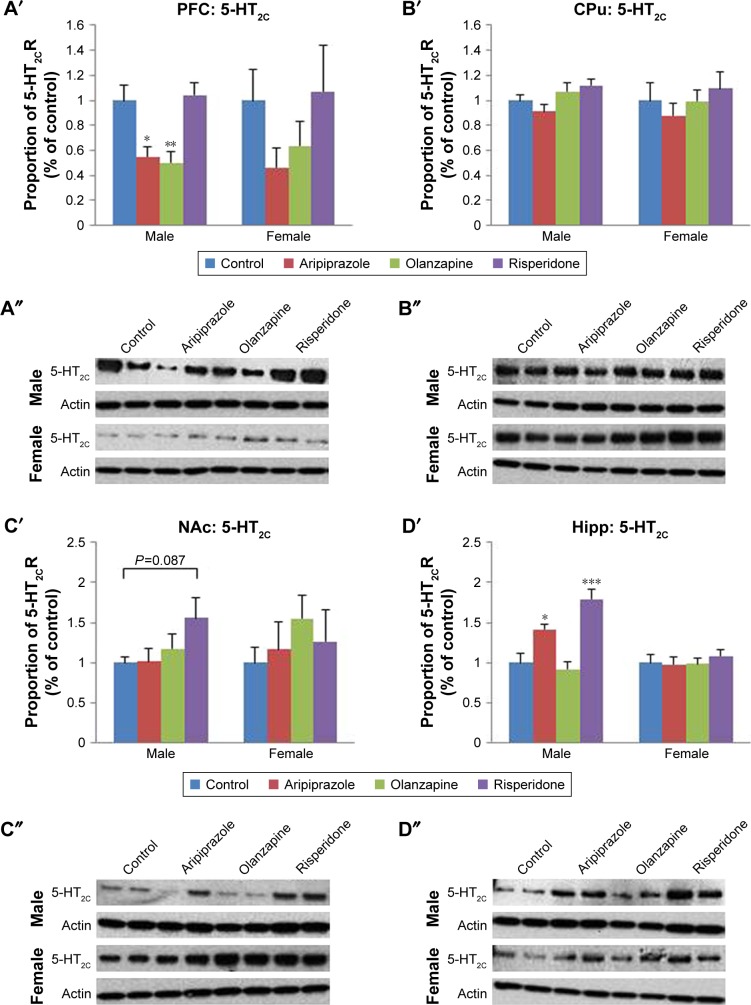
Effects of three APDs on 5-HT_2C_ expression levels in the PFC (**A**′, **A**″), CPu (**B**′, **B**″), NAc (**C**′, **C**″), and Hipp (**D**′, **D**″) of female and male rats. **Notes:** Sprague Dawley rats were treated chronically with aripiprazole (1.0 mg/kg, tid), olanzapine (1.0 mg/kg, tid), risperidone (0.3 mg/kg, tid), or control (vehicle). The number of samples per gender per group is 6. Data expressed as mean ± SEM. *P<0.05, **P<0.01, ***P<0.001 vs control. The representative bands of Western Blot are shown. **Abbreviations:** APDs, antipsychotic drugs; CPu, caudate putamen; Hipp, hippocampus; 5-HT, serotonin; NAc, nucleus accumbens; PFC, prefrontal cortex; SEM, standard error of the mean; tid, three times daily.

Analysis of 5-HT_2C_ expression via two-way ANOVA (gender × treatment) uncovered a significant effect of treatment on 5-HT_2C_ receptor protein expression in the PFC (*F*_3,44_=4.286, *P*<0.02) and hippocampus (*F*_3,45_=10.791, *P*<0.001) of rats. Additionally, a significant effect of gender was found in the hippocampus (*F*_1,45_=16.265, *P*<0.001), while a significant interaction between the factors was also found in the hippocampus (*F*_3,45_=7.511, *P*<0.001). Post hoc analysis uncovered that early treatment with both aripiprazole (−50.2%, *P*<0.05) and olanzapine (−42.5%, *P*=0.078) significantly decreased 5-HT_2C_ expression in the PFC in comparison to the control. Following analysis of the male cohort via one-way ANOVA, a significant effect of early APD treatment was discovered in the PFC (*F*_3,20_=8.004, *P*<0.01) and hippocampus (*F*_3,22_=15.474, *P*<0.001), while a trend to significant effect was found in the CPu (*F*_3,22_=2.946, *P*=0.059). Post hoc analysis found decreases in 5-HT_2C_ receptor expression in the PFC following early APD treatment with aripiprazole (−45.1%, *P*<0.05) and olanzapine (−50.1%, *P*<0.01) (Figures 4A′ and A″ and S3). Additionally, increases in 5-HT_2C_ receptor expression were uncovered in the hippocampus following early treatment with both aripiprazole (+41.5%, *P*<0.05) and risperidone (+79.6%, *P*<0.001) (Figures 4D′ and D″ and S3), while no alterations were uncovered in either the CPu (Figures 4B′ and B″ and S3) or the NAc (Figures 4C′ and C″ and S3). No significant alterations to 5-HT_2C_ receptor expression, however, were found in the female cohort.

## Discussion

The present study has, for the first time, provided insight into the long-term effects of early (juvenile) treatment with the APDs aripiprazole, olanzapine, and risperidone on the density of 5-HT receptors in the adult brain. Our investigation has revealed that juvenile APD treatment during the critical neurodevelopmental time period resulted in significant long-term alterations to 5-HT_2A_ and 5-HT_2C_ receptors, predominantly in hippocampal and cortical brain regions. Furthermore, we have uncovered more widespread alterations to the density of male 5-HT receptors in comparison to female 5-HT receptors, with changes in 5-HT_2A_ and 5-HT_2C_ receptors uncovered across multiple drug treatment groups.

While previous investigations into the long-term effects of juvenile APD use on 5-HT_1A_, 5-HT_2A_, and 5-HT_2C_ receptors have, to our knowledge, not been completed, numerous studies have examined the immediate effects of aripiprazole, olanzapine, or risperidone treatment on the density of 5-HT receptors in young^45^,^46^,^69^ and adult^65^,^67^,^70^,^71^ rodents, over both short- and long-term treatment periods. Such investigations have uncovered a trend for APD treatment to result in immediate decreases to both 5-HT_2A_ and 5-HT_2C_ receptor subtypes following a cessation of treatment. We believe that our investigation is the first to identify that if treated in a juvenile animal, this alteration to 5-HT receptor density is still prominent in adulthood.

Long-term alterations to 5-HT_2A_ receptors after early APD exposure were uncovered in hippocampal and cortical brain regions in the current study. In the male cohort, significant decreases to the density of 5-HT_2A_ receptors were found in the hippocampus of adult brains following juvenile treatment with the APDs aripiprazole, olanzapine, and risperidone. Furthermore, similar decreases were also found in the PFC of those that underwent early treatment with aripiprazole and risperidone in comparison to the control. Decreases in 5-HT_2A_ receptor densities were also found in the hippocampus of females following treatment with the APDs olanzapine and risperidone.

Although previous investigations into the immediate effects of short- and long-term treatments with olanzapine have also found region-specific alterations to the density levels of 5-HT_2A_ receptors of both young^45^,^69^ and adult^67^,^70^,^71^ rats, results from the current study extended that of previous findings. Specifically, while decreases in 5-HT_2A_ levels have been observed in the hippocampus of the present study, previous investigations over multiple time periods, and across both genders, have found no changes to hippocampal 5-HT_2A_ receptor levels in the adult brain following olanzapine treatment.^70^,^71^ Furthermore, while no changes in 5-HT_2A_ levels were uncovered in the PFC of our investigation, olanzapine has been found to immediately decrease PFC 5-HT_2A_ receptor levels in young rats following short-term treatment periods^45^,^69^ and adult rats following both short-term^70^,^71^ and long-term treatment periods.^67^ While studies on the short- and long-term effects of risperidone treatment in both young and adult rats have also uncovered similar significant decreases in 5-HT_2A_ receptor levels in the PFC,^45^,^46^,^70^ no changes in 5-HT_2A_ levels in brain regions including the CPu, NAc, and hippocampus have been previously uncovered.^45^,^69^,^70^ In addition, although significant decreases in 5-HT_2A_ receptors were observed in both the hippocampus and PFC of animals treated with aripiprazole in the present study, limited investigations have previously been completed investigating the potential for aripiprazole’s antagonistic actions on 5-HT_2A_ receptors to cause short- or long-term changes. While one investigation into the immediate effects of short-term treatment with aripiprazole has uncovered decreased levels of 5-HT_2A_ receptors in the PFC of young male rats,^45^ further investigations into the immediate and long-term effects of short- and long-term treatments will shed further light and allow further comparisons to be drawn to the current investigation findings.

In addition to the 5-HT_2A_ receptor changes, alterations to the 5-HT_2C_ receptor were found in the PFC and hippocampus of males only in the present study. Contrasting results were revealed between the aforementioned regions, with decreases in 5-HT_2C_ receptor levels uncovered in the PFC of adult male rodents following juvenile treatment with aripiprazole and olanzapine, while increases were found in the hippocampus of aripiprazole- and risperidone-treated groups in comparison to control.

Previous investigations into the immediate effects of APD treatment on 5-HT_2C_ receptor density levels have found differing results to that observed by the current study, where long-lasting effects were revealed. First, contrary to our study, alterations to 5-HT_2C_ receptors have been found across both male and female rodents in studies of varying treatment durations, utilizing both young and adult models, and through multiple brain regions. In addition, while olanzapine treatment has been found to decrease 5-HT_2C_ levels in the PFC of studies, investigations into the immediate effects of short- and long-term APD treatment on 5-HT_2C_ receptors in the hippocampus of young and adult rodents have found treatment with olanzapine resulted in either a decrease or no change in the density of receptors.^45^,^67^,^70^,^71^ In particular, while short-term olanzapine treatment of both young male and female rodents resulted in immediate decreases across cortical and striatal brain regions,^45^ variations in results have been found in the immediate effects of adult olanzapine treatment models over both short-term^70^,^71^ and long-term periods,^67^ with either decreases or no change in receptor densities found in investigated brain regions including PFC, CPu, NAc, and hippocampus. Investigations into the effects of aripiprazole and risperidone on 5-HT2C receptor have been concentrated on the immediate effects of short-term treatment periods, with no alterations to any brain regions found following short-term aripiprazole treatment across both male and female young animals^45^ or short-term risperidone treatment in adult male rodents.^70^

Minimal changes in adult 5-HT_1A_ receptors were uncovered following juvenile APD treatment in the present study. Decreases in 5-HT_1A_ receptor density levels in the adult brain were uncovered in the NAc following juvenile risperidone APD treatment in the male cohort, and in the NAc of female rodents treated with aripiprazole in comparison to the control. Previous investigations once again centered upon uncovering the immediate effect of treatment on 5-HT_1A_ receptor density levels, with the majority of studies revealing no changes in receptor levels following APD treatment with aripiprazole, olanzapine, or risperidone in investigated regions. Some short-term studies did however reveal that acute and short-term treatment in both young and adult rats increased 5-HT_1A_ receptor density levels.^46^,^70^ Specifically, while increases in 5-HT_1A_ receptor density levels have been found previously across both the PFC and hippocampus of both young and adult male rodents following short-term treatment with risperidone and olanzapine,^46^,^70^ acute treatment of female rats with aripiprazole has also uncovered increases in 5-HT_1A_ levels in the hippocampus. No alterations, however, were found following acute and long-term olanzapine or haloperidol treatments and across cortical and hypothalamic brain regions.^65^

The minimal observed alterations to the 5-HT_1A_ receptor in comparison to 5-HT_2A_ and 5-HT_2C_ may be due to a number of factors. First, of the three APDs investigated in the current study, only aripiprazole has been found to have a significant affinity for the 5-HT_1A_ receptor,^65^,^72^ while the similar antagonistic pharmacological profile of olanzapine and risperidone on 5-HT_2A_ and 5-HT_2C_ receptors may be resulting in the comparable decreases in adult brain receptor density levels between the two receptor subtypes.^22^ The 5-HT_1A_ is known to be located both pre- and postsynaptically and have autoreceptor functions.^38^,^73^,^74^ Investigations have found that the 5-HT_1A_ receptor located presynaptically in the dorsal raphe nucleus, performing regulatory functions for the 5-HT NT, and also located postsynaptically in limbic structures including the hippocampus, performing traditional postsynaptic receptor functions.^38^,^73^,^74^

The repeated antagonism of the 5-HT_2A_ and 5-HT_2C_ receptors, along with the presynaptic 5-HT_1A_ receptor, has the potential to result in a downregulation in number and sensitivity and subsequently a long-term deficiency in 5-HT NT signaling.^38^,^75^,^76^ With the 5-HT_2A_ receptor in particular well known to play critical roles in both APD treatment efficacy^19^–^22^ and the regulation and functioning of the 5-HT NT system,^36^–^38^,^77^,^78^ and with previous investigations demonstrating a correlative functions of the 5-HT NT in the pathophysiology of multiple mental illnesses,^16^,^22^,^29^,^38^,^79^,^80^ any disturbances to the regulation of the 5-HT NT system, such as through early APD treatment targeting 5-HT receptors, have the potential to alter 5-HT transmission and, thus, elicit related changes to multiple facets of mental illness over long term. Furthermore, the subsequent deficiency in projections of the 5-HT NT has been found to result in the disinhibition and therefore enhancement of the DA signal^36^–^38^ and correlated to changes in behaviors, as demonstrated in our previous investigations.^35^,^39^ Changes in behaviors including enhanced locomotor activity^36^,^37^ and anxiolytic and decreased depressive-like behaviors^8^,^36^,^75^,^81^–^84^ have previously been uncovered and correlated to the repeated antagonism of the 5-HT_2_ receptor, potentially the negatively correlated alterations to the DA signal.

APDs such as aripiprazole have also been found to elicit partial agonist effects on presynaptic 5-HT_1A_ receptors in the dorsal raphe nuclei of previously investigated brains,^76^,^85^ Investigations into the effects of APD treatment on 5-HT receptors in the dorsal raphe nuclei, however, will need to be the focus of future studies, as the focus of the current investigations only shifted to the 5-HT NT system following previous results, and thus, no relevant tissue is available for analysis.

With previous investigations into the effects of APD treatment on 5-HT receptors centered upon revealing any immediate changes in receptor density levels, and the current study looking into long-lasting effects, the contrasting results between the current and previous investigations may be the product of a myriad of influencing factors. Factors including the treatment duration of the study, differences in age of the animals treated, and duration of time between the cessation of APD treatment and detection of 5-HT receptors have previously been highlighted as having the potential to influence the observed results, with the current study specifically investigating the long-term effects of juvenile APD treatment on 5-HT receptors in the adult brain. There is the potential that the alterations to measured variables uncovered in the present study may have occurred following the cessation of APD treatment and during the drug withdrawal period. During such time, the antagonistic action of APDs on 5-HT receptors in an adult brain may result in a short-term over-compensatory increase in receptor numbers (as observed in previous investigations^65^), followed by a regulation of density over long term. Drug treatment during the critical neurodevelopmental time period may subsequently result in a long-term decrease in morphology and/or density over the large time duration, in a process previously labeled as neuronal imprinting.^25^ Previous studies have demonstrated similar age-dependent effects of drug treatment, with psychotropic drugs such as fluoxetine (a selective serotonin re-uptake inhibitor [SSRI]) previously proven to elicit different effects on a juvenile compared to an adult, mature brain.^25^,^34^,^86^,^87^

Chronologically, and as outlined briefly earlier, 5-HT is also known to play a significant role first as a trophic factor in overall brain development and then undergo significant neurodevelopmental phases itself as the NT system develops from birth through to adulthood.^8^,^31^ Specifically, the 5-HT ligand, along with the 5-HT_1A_ receptor, has been found to play key roles in overall axonal growth and synapse formation throughout the brain.^88^ Alterations to baseline levels of 5-HT during these early critical phases of neurodevelopment, through either intrinsic or extrinsic factors (eg, early APD treatment), have been found to alter the developmental trajectory of the adult brain and subsequently impact the adult brain functioning.^8^,^26^,^30^,^32^,^88^ Therefore, there is the potential that the juvenile APD treatment utilized in the present study, with a high affinity and potent actions on the 5-HT NT system, has impacted not only the observed long-lasting changes in 5-HT receptors but also widespread long-lasting alterations to overall axonal growth, neurite, and dendrite formations.

Additionally, and as indicated previously, gender-specific alterations to adult 5-HT receptor density levels were also observed following juvenile APD treatment in the present study. In particular, more widespread alterations to the 5-HT_2A_ and 5-HT_2C_ receptors were uncovered in the PFC and hippocampus of males, while minimal alterations were observed in the female cohort across all investigated 5-HT receptor subtypes and across all four brain regions and APD treatment groups. Potential influencing factors on the observed gender differences in results have been outlined extensively in previous publications.^35^,^39^ Specifically, the well-known differences in the development and expression of 5-HT receptors between genders^89^ and with the influence of the sex hormones testosterone and estrogen^26^,^82^,^89^–^91^ have the potential to play a role in the observed gender differences. Previously demonstrated gender variations to 5-HT-mediated functions have an obvious potential to influence the observed results,^89^,^92^ with the sex hormones testosterone and estrogen found to play a critical role.^89^,^91^,^93^–^96^ Changes in the levels of the sex hormone estrogen have been found to influence the levels of 5-HT ligand in brain regions including the cortex and raphe nucleus^94^–^96^ and, furthermore, alter the density levels of 5-HT_1A_ and 5-HT_2_ receptors in brain regions including the cortex, raphe nucleus, and hippocampus.^89^ Additionally, estrogen has previously been found to play a neuro-protective effect on 5-HT NT system, with studies uncovering its ability to inhibit behavioral changes in information processing mediated by both the 5-HT_1A_ and DA D_2_ receptors, an attribute that found deficient in people suffering from mental illness.^90^

## Conclusion

The current study has uncovered the potential for treatment with the APDs aripiprazole, olanzapine, and risperidone during the critical neurodevelopmental period to cause long-lasting alterations to the density of 5-HT receptors in the adult brain. In particular, significant alterations to 5-HT_2A_ and 5-HT_2C_ receptors in cortical and hippocampal brain regions were observed in the male cohort across aripiprazole, olanzapine, and risperidone APD treatment groups in comparison to controls. These observed changes are in addition to the alterations to various behavioral attributes (including anxiety and depressive-like behaviors) and the dopamine NT system (including receptors, transporters, and synthesis markers) previously reported with the same treatment model in our laboratory.^35^,^39^ Although the observed alterations to the 5-HT NT system in the investigated regions provide some evidence of the potential for early APD treatment to elicit long-term alterations to a NT system functioning, further investigations have the potential to uncover both the scope of changes elicited on the 5-HT NT system and potential alterations to other NT groups. Other NT systems including the adrenergic and muscarinic NT systems are also known to be a part of the pharmacological mechanisms of action of APDs and interplay with other NT systems,^37^,^97^–^99^ and thus, antagonist actions during the critical neurodevelopmental time period have the potential to elicit long-lasting changes that may be exhibited clinically. Furthermore, investigations into the effects of juvenile APD in a disease animal model would also provide invaluable insight into the potential long-lasting effects of treatment during such a critical neurodevelopmental time period. The alterations observed in the present study provide some of the first evidence of the potential of juvenile APD treatment with aripiprazole, olanzapine, and risperidone to elicit long-term alterations to the 5-HT NT system in the adult brain. With all three APDs approved for use in adolescents with various medical conditions and also known to be prescribed off-label, the potential long-term effects of early use should be highlighted before they are prescribed clinically, especially in the male cohort where the vast majority of alterations have been found.
